# Effect of Ishophloroglucin A, A Component of *Ishige okamurae*, on Glucose Homeostasis in the Pancreas and Muscle of High Fat Diet-Fed Mice

**DOI:** 10.3390/md17110608

**Published:** 2019-10-25

**Authors:** Hye-Won Yang, Myeongjoo Son, Junwon Choi, Seyeon Oh, You-Jin Jeon, Kyunghee Byun, BoMi Ryu

**Affiliations:** 1 Department of Marine Life Science, School of Marine Biomedical Sciences, Jeju National University, 1 Ara 1-dong, Jejudaehak-ro, Jeju 63243, Korea; koty221@naver.com (H.-W.Y.); youjinj@jejunu.ac.kr (Y.-J.J.); 2 Department of Anatomy & Cell Biology, Gachon University College of Medicine, Incheon 21936, Korea; mjson@gachon.ac.kr (M.S.); choijw88@gc.gachon.ac.kr (J.C.); 3 Functional Cellular Networks Laboratory, College of Medicine, Department of Medicine, Graduate School and Lee Gil Ya Cancer and Diabetes Institute, Gachon University, Incheon 21999, Korea; seyeon8965@gachon.ac.kr; 4 Marine Science Institute, Jeju National University, Jeju 63333, Korea

**Keywords:** functional food, diabetes, *Ishige okamurae*, Ishophloroglucin A

## Abstract

Ishophloroglucin A (IPA), a component of *Ishige okamurae* (IO), was previously evaluated to standardize the antidiabetic potency of IO. However, the potential of IPA as a functional food for diabetes prevention has not yet been evaluated. Here, we investigated if 1.35 mg/kg IPA, which is the equivalent content of IPA in 75 mg/kg IO, improved glucose homeostasis in high-fat diet (HFD)-induced diabetes after 12 weeks of treatment. IPA significantly ameliorated glucose intolerance, reducing fasting glucose levels as well as 2 h glucose levels in HFD mice. In addition, IPA exerted a protective effect on the pancreatic function in HFD mice via pancreatic β-cells and C-peptide. The level of glucose transporter 4 (GLUT4) in the muscles of HFD mice was stimulated by IPA intake. Our results suggested that IPA, which is a component of IO, can improve glucose homeostasis via GLUT4 in the muscles of HFD mice. IO may be used as a functional food for the prevention of diabetes.

## 1. Introduction

Seaweeds contain bioactive substances, such as polysaccharides, proteins, lipids, and polyphenols, and have been reported to have nutraceutical and pharmaceutical potential in functional foods [1,2]. *Ishige okamurae* (IO), an edible seaweed, possesses bioactive substances, such as ishophloroglucin A (IPA), diphlorethohydroxycarmalol (DPHC), and fucoxanthin, as well as other secondary metabolites [3]. In our previous study, we suggested that IPA could be used to standardize the antidiabetic activity potency of IO extract in vitro [4]. However, the use of IPA in functional foods for diabetes prevention in vivo, in high-fat diet (HFD)-fed mice, has not yet been determined.

Diabetes mellitus is a chronic metabolic disorder caused by the inadequate balance of glucose homeostasis [5]. Glucose homeostasis is maintained by the tight regulation of blood glucose by insulin and glucagon [6]. In particular, glucose transporters (GLUT), with substrate specificities that dictate their functional roles, regulate glucose level both outside and inside of the cell [7]. Most of the current drugs for the treatment of diabetes aim to improve insulin production and metabolic regulation. Furthermore, previous studies have focused on the prevention of diabetes for both type 1 and type 2 diabetes [8,9]. In addition, the improper balance of glucose homeostasis can be prevented or reduced by functional foods. Therefore, there has recently been much interest in the use of natural products as a source of stronger and safer antidiabetic therapies [10,11,12].

Previous studies have reported that complementary and alternative natural products, such as herbal therapies, have been used for the prevention and/or treatment of diseases, such as diabetes, hypertension, and cardiovascular disease [13,14]. Increased intake of high-calorie and high-fat diets and decreased physical activity have led to a high rate of chronic diseases, such as heart diseases, diabetes, and hypertension [15]. Health is recognized by many as an important personal and social value; consumers have become increasingly interested in the benefits of food that can help achieve or maintain a healthy lifestyle [16,17]. Previous studies have reported that adequate nutrition is an essential aspect of diet and chronic diseases, as well as influencing a person’s health status [18,19]. In addition, the studies have shown that health is an important motivation for nutraceutical consumption [20].

Therefore, this study evaluated the effect of IPA on glucose transporters, such as GLUT2 and GLUT4, which are involved in glucose homeostasis. In addition, we examined if their related transcription factors in the pancreas and muscle could regulate the blood glucose level in HFD mice. The data from this study address the effect of IPA on glucose homeostasis in the pancreas and muscle of HFD mice, and shed further light on the strategic potential of IPA as a functional food for the improvement of diabetes in the future.

## 2. Results

### 2.1. Improvement in Glucose Tolerance by IPA in HFD Mice

**Figure 1:**
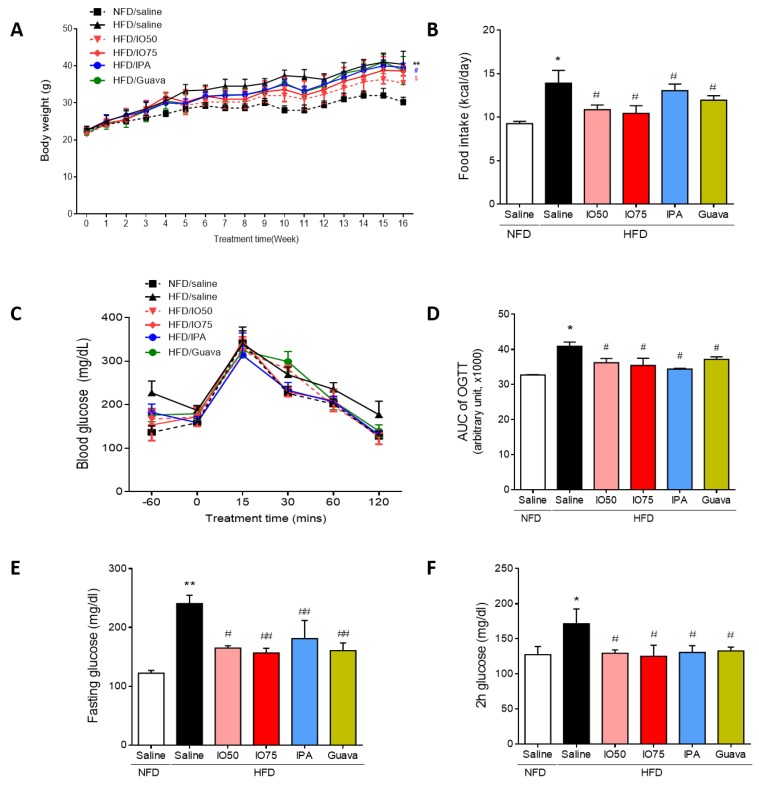
Effect of Ishophloroglucin A (IPA) on serum glucose level in high fat diet-fed mice model. The mice receiving 45% high fat diet for 8 weeks exhibit improved glucose tolerance but, IPA oral administration reduced glucose tolerance. (**A**) Body weight and (**B**) food intake were measured weekly during 14 weeks on different diets/treatments. (**C**,**D**) Oral glucose tolerance tests (OGTT) were measured and calculated area under the curve (AUC) from the GTT in all mice groups. (**E**) Fasting and (**F**) feeding glucose levels were in all mice group. Data are expressed as mean ± S.D. * *p* < 0.05 or ** *p* < 0.01 vs. NFD/saline; ^#^ *p* < 0.05, ^##^ *p* < 0.01 vs. HFD/saline; ^$^ *p* < 0.05 vs. HFD/Guava.

To assess whether the increased metabolic disorders in HFD mice were improved by IPA, we investigated the body weight, food intake, and fasting and feeding glucose levels in HFD mice. The concentration of IPA used in this experiment was the equivalent content of IPA (1.35 mg/kg) in IO extract (75 mg/kg). As shown in Figure 1A, the body weight of HFD mice was not different at the beginning of the study between groups. After 4 weeks of the HFD/IO treatment, we fed mice IO extract (50 and 75 mg/kg, HFD/IO), IPA (1.35 mg/kg, HFD/IPA), and guava (75 mg/kg, HFD/Guava), used as positive control for blood glucose level control in diabetes [21], for 12 weeks. After 12 weeks, the HFD group gained more body weight at each time point compared with the NFD group. However, the HFD/IPA (1.35 mg/kg) group showed a significantly lower increased in body weight compared with the HFD group. In addition, the amount of HFD consumed is required to maintain a constant energy intake [22]. A previous study has reported that HFD may be overconsumed in an attempt to derive the necessary levels of energy [23]. The HFD group had a higher food intake than the NFD group (Figure 1B). The increased food intake of mice fed the HFD was significantly decreased by IO (50 and 75 mg/kg), IPA (1.35 mg/kg), and guava (75 mg/kg).

To investigate the effect of IPA on glucose tolerance, oral glucose tolerance tests (OGTTs) were conducted at different time points (30, 60, 90, and 120 min) after 12 weeks of HFD/IO, HFD/IPA, and HFD/Guava treatment. As shown in Figure 1C, HFD groups showed a lower glucose tolerance, with higher glucose level after 120 min, compared with the NFD group. However, the increased glucose levels were significantly reversed by HFD/IO (50 and 75 mg/kg), HFD/IPA (1.35 mg/kg), and HFD/Guava (75 mg/kg). To evaluate the degree of the glucose tolerance impairment, we calculated the area under the glucose curve (AUC) in the OGTT in each group. In Figure 1D, the AUC of OGTT in the HFD group was significantly increased compared with that in the NFD group. The AUC of OGTT in the HFD/IO, HFD/IPA and HFD/Guava groups was significantly decreased compared with that in the HFD group. Fasting glucose and 2 h glucose levels play a role in maintaining glucose homeostasis in diabetes. As shown in Figure 1D,E, fasting and 2 h glucose levels were significantly increased in the HFD group compared with that in the NFD group. Increased fasting and 2 h glucose levels were significantly decreased by HFD/IO (50 and 75 mg/kg), HFD/IPA (1.35 mg/kg), and HFD/Guava (75 mg/kg) treatments. These data suggested that IPA and IO containing IPA can improve glucose tolerance in HFD mice. We further evaluated the maintenance of glucose homeostasis in the blood of HFD mice as a means to control blood glucose level.

### 2.2. Regulation of Glucose Homeostatic Imbalance by IPA in HFD Mice

**Figure 2:**
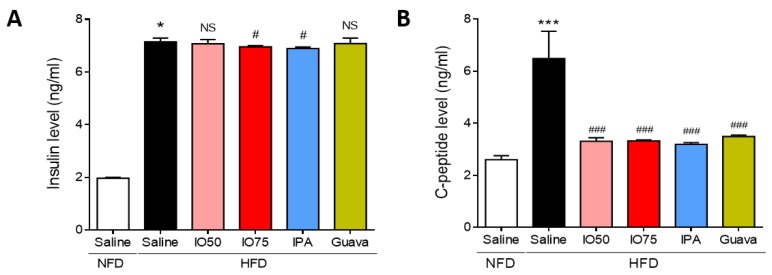
Effect of IPA on insulin and C-peptide level in high fat diet-fed mice model. (**A**) Insulin and (**B**) C-peptide levels were measured by ELISA kit in all mice groups. Data are expressed as mean ± S.D. * *p* < 0.05 or *** *p* < 0.001 vs. NFD/saline; ^#^ *p* < 0.05, ^###^ *p* < 0.001 vs. HFD/saline. NS, not significant.

Next, we assessed whether the activation of the insulin and C-peptide level in the blood of HFD mice was regulated by HFD/IPA to control glucose metabolism. As shown in Figure 2A, the insulin level was significantly higher in HFD mice than in NFD mice, indicating that insulin resistance was induced in HFD mice. However, the insulin level was significantly lower in the HFD/IO (75 mg/kg) and HFD/IPA (1.35 mg/kg) groups than in HFD mice. As shown in Figure 2B, the C-peptide level was significantly higher in HFD mice than in NFD mice, indicating that the pancreas produced insulin to regulate glucose homeostasis. In contrast, the increase in C-peptide level was markedly decreased by HFD/IO (50 and 75 mg/kg), HFD/IPA (1.35 mg/kg), and HFD/Guava (75 mg/kg) treatment. These results showed that the decrease in insulin and C-peptide levels by IPA and IO containing IPA could regulate glucose homeostasis in HFD-induced insulin resistance.

### 2.3. Protective Effect of IPA on Pancreatic Dysfunction in HFD Mice

**Figure 3:**
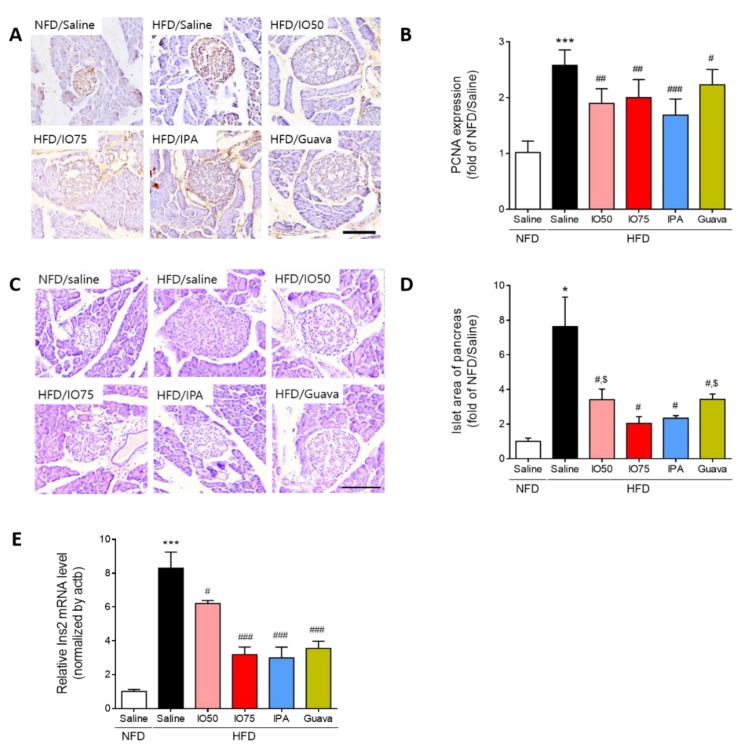
Effect of IPA on the morphology and area of pancreatic islets in high fat diet-fed mice model. (**A**) Proliferative cell marker (Proliferating cell nuclear antigen, PCNA) was detected by immunohistochemistry and (**B**) quantitative graph shows the level of PCNA expression on pancreatic islet from representative images. (**C**) Hematoxylin and eosin (H&E) stained histological images show islets of pancreas (round shape and pale color) and (**D**) pancreatic islet size were measured by image j software from representative images. (**E**) The expression level of Ins2 was measured by qRT-PCR. Scale bar = 100 µm, Data are expressed as mean ± S.D. * *p* < 0.05, or *** *p* < 0.001 vs. NFD/saline; ^#^ *p* < 0.05, ^##^ *p* < 0.01, or ^###^ *p* < 0.001 vs. HFD/saline; ^$^ *p* < 0.05 vs. HFD/Guava.

To examine whether the increased number of pancreatic islet cells in HFD mice was reduced by IPA and IO for the protection of β-cell function, we investigated the effect of IPA on the change in morphology and the area of pancreatic islets in HFD mice by using immunohistochemistry and hematoxylin and eosin (H & E) staining. The proliferating cell nuclear antigen (PCNA), a proliferative cell marker, was detected to examine the proliferation of pancreatic islet cells [24]. As shown in Figure 3A,B, we measured the intensity of PCNA staining in representative images to quantify the effect of IPA and IO on the proliferation of β-cells in the pancreatic islets. The PCNA intensity in pancreatic islet cells in HFD mice was significantly higher than in NFD mice. However, the increased PCNA intensity was significantly decreased in the HFD/IO, HFD/IPA, and HFD/Guava groups. As shown in Figure 3C,D, the size of pancreatic islets from the histological changes in HFD mice was markedly elevated compared with that in NFD mice, whereas the size of pancreatic islets was significantly reduced in the HFD/IO, HFD/IPA, and HFD/Guava groups. These data suggested that IPA and IO containing IPA could protect against pancreatic dysfunction that occurs in HFD-induced diabetes.

Next, we examined Ins2 mRNA expression in the pancreas of HFD mice by using qRT-PCR to evaluate β-cell function by HFD/IPA. As shown in Figure 3E, Ins2 mRNA expression was markedly increased in HFD mice compared with that in NFD mice. However, the Ins2 mRNA expression was significantly decreased in the HFD/IO, HFD/IPA, and HFD/Guava mice. Interestingly, 1.35 mg/kg HFD/IPA, which is the equivalent content of IPA in IO, reduced Ins2 mRNA, as well as 75 mg/kg HFD/IO. These results suggested that the decrease in Ins2 mRNA level induced by IPA was able to regulate the elevated blood glucose level in HFD mice.

### 2.4. Effect of IPA on Glucose Transport in the Pancreas and Skeletal Muscle of Mice

**Figure 4:**
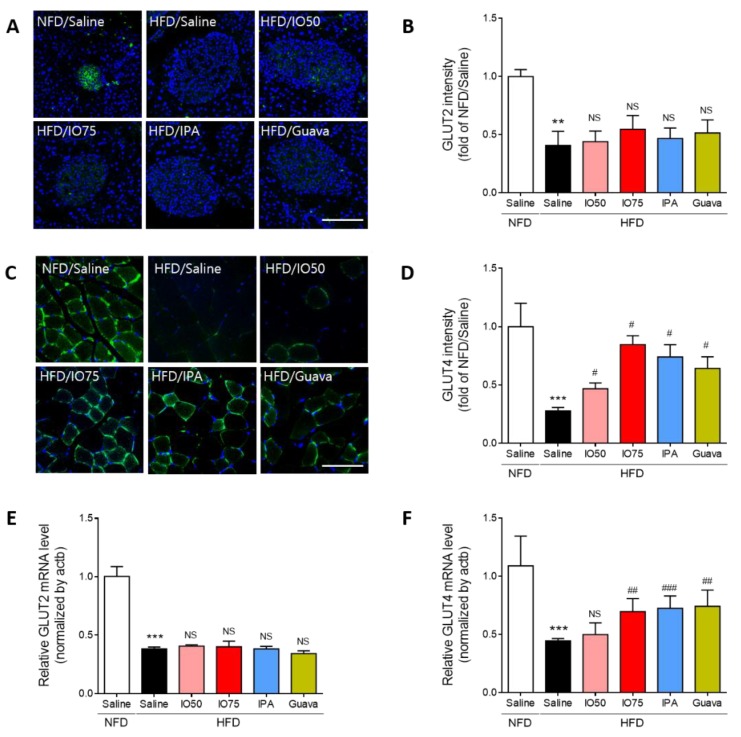
Effect of IPA on GLUT2 and GLUT4 expression increase of pancreas and skeletal muscle in high fat diet-fed mice model. (**A**) Confocal microscopic images show GLUT2 expression and GLUT2 (green) and nuclei (blue; DAPI) were detected of pancreas tissue of mice. (**B**) Quantitative graph was show level of GLUT2 expression from representative images and Zen 2012 software was used for measurement. (**C**) Confocal microscopic images show GLUT4 expression and GLUT4 (green) and nuclei (blue; DAPI) were detected of skeletal muscle tissue of mice. (**D**) Quantitative graph was show level of GLUT4 expression from representative images and Zen 2012 software was used for measurement. (**E**,**F**) The expression levels of GLUT2 and GLUT4 were measured by qRT-PCR. Scale bar = 100 µm, Data are expressed as mean ± S.D. ** *p* < 0.01, or *** *p* < 0.001 vs. NFD/saline; ^#^ *p* < 0.05, ^##^ *p* < 0.01, or ^###^ *p* < 0.001 vs. HFD/saline. NS, nor significant.

The glucose transporter 2 (GLUT2) is an essential agent in the induction of glucose-stimulated insulin secretion and glucose tolerance in the β-cells of the pancreas [25]. Thus, we evaluated the effect of IPA on GLUT2 expression in the pancreas of HFD mice by using immunofluorescence, as shown in Figure 4A,B. The intensity of GLUT2 in HFD mice was significantly decreased compared to that in NFD mice. After the treatment with IO (50 and 75 mg/kg), IPA (1.35 mg/kg), and Guava (75 mg/kg), no significant decrease in the intensity of GLUT2 in HFD mice was found compared with that in HFD mice.

Previous studies reported that the defects of glucose transporter 4 (GLUT4) translocation were caused by impairment of glucose metabolism in HFD mice [26]. Thus, we examined the effect of IPA on the translocation of GLUT4 in the membrane of muscles in HFD mice by using immunofluorescence, as shown in Figure 4C,D. HFD/IO mice showed a slight increase in GLUT4 intensity, with a significant increase in the 75 mg/kg HFD/IO group. HFD/IPA and HFD/Guava treatment resulted in significantly increased GLUT4 translocation. These results showed that the increase in GLUT4 by IPA could improve glucose metabolism in the skeletal muscle of HFD mice.

Interestingly, the transcription of GLUT2 and GLUT4 is known to regulate glucose sensing and glucose transporters [27]. We measured the mRNA expression of GLUT2 and GLUT4 in the pancreas and skeletal muscle of HFD mice by qRT-PCR to evaluate glucose metabolism by IPA. As shown in Figure 4E,F, the mRNA expression of GLUT2 and GLUT4 was significantly decreased in HFD mice compared with that in NFD mice. The decrease in mRNA expression of GLUT2 was not significantly different in the IO (25 and 75 mg/kg), IPA (1.35 mg/kg), and Guava (75 mg/kg) treatment groups. However, the decrease in the mRNA expression of GLUT4 was significantly increased by the IO (25 and 75 mg/kg), IPA (1.35 mg/kg), and Guava (75 mg/kg) treatments. These results indicated that the increase in GLUT4 expression by IPA can improve glucose transport, thereby controlling glucose homeostasis, in HFD mice.

## 3. Discussion

A previous study reported that IPA isolated from IO and the equivalent content of IPA in IO extract showed an antidiabetic activity similar to that of IO extract [4]. However, the effect of IO, including IPA, as an agent for diabetes prevention has not yet been assessed on glucose homeostasis in the pancreas and muscles of HFD mice. In this study, our data suggest that IPA can improve glucose homeostasis in the pancreas and muscle, resulting in the regulation of blood glucose level in HFD-induced diabetes.

The HFD mouse is a robust and efficient model for type 2 diabetes and can be used for mechanistic studies [28]. Type 2 diabetes is a metabolic disorder characterized by insulin resistance and pancreatic β-cell dysfunction [29]. Insulin and C-peptide are present in the pancreas at equivalent concentrations [30]. C-peptide promotes the activation of the insulin receptor and increases glycogen synthesis via the insulin signaling pathway, but it does not affect glucose lowering [31,32]. It is also suggested that C-peptide may have a suppressive effect on insulin action in type 2 diabetes [31]. IPA significantly decreased the insulin and C-peptide levels in the plasma of HFD mice, suggesting the protective action of IPA against pancreatic dysfunction in HFD-induced diabetes.

The maintenance of glucose homeostasis is regulated by insulin secretion from pancreatic β-cells and hepatic glucose production, as well as glucose disposal into the muscle and adipose tissue [33]. The failure of β-cell function in pancreas is associated with HFD-induced diabetes [34]. Previous studies reported that the decrease in pancreatic β-cell mass was a common characteristic of subjects with diabetes [35,36]. In addition, in HFD mice, type 2 diabetes occurs when β-cells in the pancreas fail to secrete sufficient amounts of insulin to meet the metabolic demand [33]. The response of the elevated glucose levels in pancreatic β-cells was regulated by an increase in insulin hormone production and secretion. However, the prolonged stimulation of pancreatic β-cells by increased blood glucose levels results in β-cell function failure and insulin release reduction [37,38]. We confirmed that IPA treatment reduced β-cell function failure in HFD mice.

Hyperglycemia and glucose intolerance were improved by glucose transporters such as GLUT2 and GLUT4; their function is to regulate glucose level both outside and inside the cell [7,39]. In addition, glucose transport occurs in pancreatic β-cells, muscles, adipose tissue, and the brain to maintain glucose homeostasis and metabolic harmony [40]. GLUT4 plays a key role in the skeletal muscle, suppressing glucose intolerance and insulin resistance. The skeletal muscle is a major organ in insulin-mediated GLUT4 translocation for glucose metabolism [41]. The GLUT2 level in the pancreas was not changed by IPA treatment, whereas the GLUT4 level in the muscle was increased by IPA treatment in HFD mice. This indicated that IPA improved glucose homeostasis in HFD-induced diabetes via GLUT4 translation.

## 4. Materials and Methods

### 4.1. Preparation of IO and IPA

A 50% ethanol extract of IO was provided by Shinwoo Co. Ltd. (Lot No. SW9E29SA, Gyeonggi-do, Korea). Briefly, the IO used in this study was standardized on the assumption of diphlorethohydroxycarmalol (DPHC, 2.37%) by an HPLC analysis method [4] with a slight modification. IPA was purified on a pure C-850 FlashPrep chromatography system equipped with a PDA detector or an ELSD detector (all from Buchi, Flawil, Switzerland), equipped with an YMC Pack ODS-A (20 mm × 250 mm, 5 µm). The mobile phase consisted of (A) 0.1% formic acid in water and (B) ACN containing 0.1% formic acid. The HPLC elution was conducted as follows: 20%–40% B for 25 min, followed by a 10 min re-equilibration period of the column. The flow rate was maintained at 9 mL/min and the injection volume was 2 mL. IPA was verified by using quadrupole time-of-flight liquid chromatography-mass spectrometry (Q-TOF LC-MS/MS) using an electrospray ionization (ESI) source (maXis-HD, Bruker Daltonics, Breman, Germany) at the Korea Basic Science Institute (KBSI; Ochang, South Korea), targeted at the m/z 1986.26 fragment.

### 4.2. Animals

Six-week-old C57BL/6N male mice were used in this research. Animals were acclimated for 1 week. After acclimation, the mice were fed a 45% high-fat diet (HFD; Research Diets, Inc., New Brunswick, NJ, USA) for 8 weeks, except for the control group. After 4 weeks, the mice were allocated into six treatment groups: NFD/Saline, HFD/Saline, HFD/IO50, HFD/IO75, HFD/IPA, HFD/Guava. Each group was orally administrated saline and IO50 (50 mg/kg/day), IO75 (75 mg/kg/day), IPA (1.35 mg/kg/day) for 4 weeks after the separation. During the 8 weeks, the body weight, fasting glucose test, and feeding glucose test was measured once every week. An oral glucose tolerance test (OGTT) was performed once before the animals were sacrificed and the data were analyzed through the calculation of the area under curve (AUC). The animal protocol for the experiments was approved by the Animal Center of Lee Gil Ya Cancer and the Diabetes Institute of Gachon University. All experiments conformed to the AAALAC international guidelines and veterinary advice. The number of this study is LCDI-2018-0112.

### 4.3. Insulin Measurement

The rat/mouse insulin ELISA Kit (Millipore, Burlington, MA, USA) was used to measure the concentration of insulin in the blood. The blood serum was collected before the animals were sacrificed and then centrifuged at 2,000 g for 10 min. The supernatant and serum were transferred to the new tube and used for this study. The absolute concentration of insulin (Abcam, San Francisco, CA, USA) was measured by using an ELISA kit. The absorbance of the solution at 450 nm was measured by using an ELISA plate reader (Molecular Devices, San Jose, CA, USA).

### 4.4. C-peptide Measurement

A mouse C-peptide ELISA kit (KAMIYA Biomedical Company, Tukwila, WA, USA) was used to measure the absolute concentration of C-peptide in the blood. The absorbance of the solution at 450 nm was measured by using an ELISA plate reader (Molecular Devices, Suunyvale, CA, USA).

### 4.5. Sample Preparation

#### 4.5.1. RNA Isolation and cDNA Synthesis

Visceral fat tissues were homogenized by the protocol innated in MACS (Miltenyi Biotec, Bergisch Gladbach, Germany) machine with 500 µL of RNisol (TAKARA, Tokyo, Japan). The supernatant of the tissue lysates was transferred to the new tube and 100 µL of chloroform was added. After vortex mixing for 3 s and incubation on ice for 10 min, the lysates were centrifuged at 12,000 g for 15 min at 4 °C. Remove the supernatant gently. RNA pellets were washed in 70% ethanol (EtOH), then centrifuged at 12,000 g for 15 min at 4 °C. The RNA pellet was dissolved in 40 µL of diethyl pyrocarbonate water (DEPC), and 1 µg of RNA was synthesized into complimentary DNA by using Prime Script 1st-strand cDNA Synthesis Kit (TAKARA, Shiga, Japan).

#### 4.5.2. Tissue Preparation

Visceral fat tissue was fixed in 4% paraformaldehyde (PFA) solution to fix for 1 week. The fixed tissue was washed for 10 min in running water. To prepare a paraffin block, the tissues were incubated in the tissue processor machine (Shandon Citadel, Ramsey, MN, USA) for 14 h. Subsequently, after the machine process was finished, the tissues were embedded in the cassette with the paraffin. Blocks were stored at room temperature.

### 4.6. Quantitative Real Time Polymerase Chain Reaction (qRT-PCR)

To validate the gene expression of each factor, 1 µg of cDNA was used with the primers (Supplementary Table S1.) and distilled water. The solution was added 10 µL of cyber green solution (CYBG, TAKARA, Mountain View, CA, USA). The mixed solution was distributed in a 384-well plate (Bio-RAD, Hercules, CA, USA). CFX386 touch (Bio-Rad, Hercules, California, USA) was used to perform the qRT-PCR. The reaction efficiency and the number of cycles were determined by innate software.

### 4.7. Immunohistochemistry: 3, 3 -Diaminobenzidine (DAB) Staining

Paraffin-sectioned slides were deparaffinized with xylene for 5 min twice times. And then, the slides were incubated in antigen-retrieval solution for 5 min in the microwave. The slides were washed in running water for 5 min, and blocking solution was added to the tissue, depending on the host, for 1 h. After three washes in PBS, for 5 min each, the slides were incubated with the primary antibody (Supplementary Table S2.) overnight. Proliferating cell nuclear antigen (PCNA, Abcam) primary antibody was diluted to 1:100 with blocking solution. The slides were washed three times with PBS solution, for 5 min each, and secondary antibody was added to the slides. Biotinylated secondary antibody were diluted to 1:200 with PBS for 2 h. To amplify the signal, ABC kit (Vector Laboratories Inc, Burlingame, CA, USA) was used for 30 min (dilution 1:50 in PBS). The slides were washed three times in PBS, for 5 min each, and then developed by the application of DAB solution until signal was visible. After development, the slides were washed with running water for 10 min and mounted with DPX solution (Sigma-Aldrich, St. Louis, MO, USA). Images were taken by light microscopy (Olympus, Tokyo, Japan). To measure the intensity of DAB staining, ImageJ software (NIH, Bethesda, MD, USA) was used.

### 4.8. Immunohistochemistry: Fluorescence Staining

Paraffin-sectioned slides were deparaffinized twice with xylene for 5 min. After serial incubation in EtOH, the slides were incubated in antigen-retrieval solution for 5 min in the microwave. The slides were washed in running water for 5 min and then incubated in blocking solution, depending on the host, for 1 h. After three washes in PBS, for 5 min each, the slides were incubated with the primary antibody overnight. Glut2 (Santa Cruz Biotechnology Inc, Santa Cruz, CA, USA) and Glut4 (Santa Cruz) primary antibody were diluted to 1:100 with blocking solution. The slides were washed with 0.1% Triton X-100 in PBS (TPBS), and then incubated with 488-fluorescent anti-mouse secondary antibody (Abcam) solution 2 h. The secondary antibody was diluted 1:200. After incubation for 2 h in the dark at room temperature, the slides were washed three times with TPBS for 10 min. The slides were mounted by using with Vectashield (Vector Laboratories Inc, Burlingame, CA, USA) and images were taken by using a confocal laser microscope (LSM 710, Carl Zeiss, Oberkochen, Germany). To measure the intensity of the immunofluorescence staining, ImageJ software (NIH) was used.

### 4.9. Histology: Hematoxylin and Eosin Staining

Paraffin-sectioned slides were deparaffinized twice with xylene for 5 min. After serial incubation in EtOH and running water to rehydrate, the slides were stained with hematoxylin (DAKO, Carpinteria, CA, USA) for 2 min at room temperature. The slides were washed with running water for 5 min, stained with eosin (Sigma-Aldrich) for 7 s, and washed with running water for 5 min. The slides were mounted by using DPX (Sigma-Aldrich) solution and viewed by light microscopy. The size of visceral fat was measured by using ImageJ (NIH).

### 4.10. Statistics

The Kruskal–Wallis test and the Mann–Whitney U post-hoc test were used to compare the statistical differences between the groups, computed by using SPSS version 22 (IBM Corporation, Armonk, NY, USA). The results were presented as the mean ± standard deviation (SD). Differences were considered significant at * *p* < 0.05 vs. NFD/Saline; ** *p* < 0.01 vs. NFD/Saline, *** *p* < 0.001 vs. NFD/Saline; ^#^ *p* < 0.05 vs. HFD/Saline; ^##^ *p* < 0.01 vs. HFD/Saline; ^###^ *p* < 0.001 vs. HFD/Saline.

## 5. Conclusions

Collectively, we demonstrated the effect of IPA on glucose homeostasis in the pancreas and muscles of mice with HFD-induced diabetes and determined potential strategies to ameliorate metabolic disorders in patients with diabetes in the future. IO, containing IPA, may be used to develop functional foods for diabetes prevention.

## Supplementary Materials

The following are available online at https://www.mdpi.com/1660-3397/17/11/608/s1, Table S1: List of primer for quantitative polymerase chain reaction (qRT-PCR), Table S2: List of anti-body for immunohistochemistry.
