# Supplementary material for: Effect of Ishophloroglucin A, A Component of Ishige okamurae, on Glucose Homeostasis in the Pancreas and Muscle of High Fat Diet-Fed Mice
Source: Mar Drugs. 2019 Oct 25;17(11):608. doi: 10.3390/md17110608 (PMC6891760; doi:10.3390/md17110608)
Supplement: Supplementary file 1 [file marinedrugs-17-00608-s001.zip › marinedrugs_supplementary materials.docx]

**Table S1: List of primer for quantitative polymerase chain reaction (qRT-PCR)**

| **Gene** | | **Primers** |  |
| --- | --- | --- | --- |
| *β-actin* | Forward | 5'-ACA AAG CTG TTC AGT GTC TCC A-3’ | |
|  | Reverse | 5'-CTC CGT TTC CAG AAT ACA CAC A-3’ | |
| *Insulin2* | Forward | 5'-CTA GCA AGG CTC AAT TCC ATC T -3’ | |
|  | Reverse | 5'-ACA CTG GCT AGT CAT TGG TCC T -3’ | |
| *Glut2* | Forward | 5'-CTA GCA AGG CTC AAT TCC ATC T -3’ | |
|  | Reverse | 5'-ACA CTG GCT AGT CAT TGG TCC T-3’ | |
| *Glut4* | Forward | 5'-CTG TAT TCT CAG CTG TGC TTG G -3’ | |
|  | Reverse | 5'-TCT GTT CAA TCA CCT TCT GTG G -3’ | |

**Table S2 : List of anti-body for immunohistochemistry**

| **Anti-body Company** | | **dilution rate** |  |
| --- | --- | --- | --- |
| *PCNA* | Abcam  (ab2426) | 1: 100 | |
| *Insulin2* | Santa Cruz | 1: 100 | |
|  | (sc-52037) |  |  |
| *Glut2* | Santa Cruz | 1: 100 | |
|  | (sc-9117) |  |  |
| *Glut4* | Santa Cruz | 1: 100 | |
|  | (sc-53566) |  |  |
